# The effects of three metaphylactic drugs on the nasal prevalence of *Pasteurellaceae* throughout the feeding period

**DOI:** 10.1093/tas/txag171

**Published:** 2026-10-23

**Authors:** Ethan P Dudley, John D Loy, P Rand Broadway, Robert Valeris-Chacin, Kristin E Hales

**Affiliations:** Department of Veterinary Pathobiology, Texas A&M University, 3201 Russell Long Blvd, Canyon, TX 79015, United States; Nebraska Veterinary Diagnostic Center, School of Veterinary Medicine and Biomedical Sciences, University of Nebraska-Lincoln, 4040 E Campus Loop N, Lincoln, NE 68583, United States; United States Department of Agriculture, Agricultural Research Service, Livestock Issues Research Unit, 1604 East FM 1294, Lubbock, TX 79403, United States; Department of Veterinary Pathobiology, Texas A&M University, 3201 Russell Long Blvd, Canyon, TX 79015, United States; Department of Animal and Food Sciences, Texas Tech University, 1308 Indiana Ave, Lubbock, TX 79415, United States

**Keywords:** metaphylaxis, beef cattle, *Pasteurellaceae*, MALDI-TOF, nasal prevalence

## Abstract

Bovine Respiratory Disease (BRD) is a substantial cause of morbidity in cattle feedlots. Antimicrobial metaphylaxis is widely used to prevent disease and improve cattle growth performance, but its effects on the isolation of BRD-associated bacteria are not well defined. The aim of this study was to quantify the effects of three common metaphylactic drugs on the isolation frequency of members of the *Pasteurellaceae* throughout the feeding period. Upon feedlot arrival, cattle at high risk of developing BRD (*n* = 246) were randomized into four treatment groups: control, florfenicol, ceftiofur, and tulathromycin. Deep nasal swabs were collected upon arrival, approximately 1 month on feed, and prior to harvest (∼245 days on feed). Bacteriological culture was performed on all swabs, and colonies with morphology consistent with BRD-associated bacteria were subjected to MALDI-TOF MS for final confirmation. Nasal shedding prevalence over time was modeled with GEE multinomial logistic regression. An overall metaphylaxis effect was observed (*P* < 0.01). *Pasteurellaceae* nasal shedding prevalence did not differ significantly between experimental groups at one month on feed. By contrast, florfenicol-treated cattle had a greater proportion of samples without isolation than ceftiofur-treated cattle (10% versus 2%, *P* < 0.05). At the end of the feeding period, tulathromycin- and florfenicol-treated cattle had the lowest proportion of *Pasteurellaceae* isolates (28% and 32%, respectively, *P* < 0.05). Taken together, our findings highlight the differing effects of metaphylactic drugs in decreasing the nasal prevalence of bacterial pathogens in high-risk feedlot cattle.

## Teaser

This research showed that metaphylaxis could have a lasting impact on bacterial nasal prevalence in high-risk feedlot cattle.

## Introduction

Bovine Respiratory Disease (BRD) is a substantial source of morbidity and mortality among feedlot calves, with respiratory disease-related death losses estimated to be approximately $907.8 million annually in the cattle industry (Peel 2020). BRD is a complex, multifactorial disease thought to be caused by the interaction among opportunistic bacterial pathogens, respiratory viruses, and stressors such as weaning and transportation (Griffin et al. 2010; Taylor et al. 2010). Four bacteria are consistently isolated or detected from cattle with clinical signs of BRD: *Pasteurella multocida, Mannheimia haemolytica, Histophilus somni*, and *Mycoplasma bovis*, with *M. haemolytica* being most frequently isolated from the lungs of cattle from BRD associated mortality (Klima et al. 2014).

Although it has been shown that BRD mortalities have distinct pathological characteristics based on the primary microbiological agent (Booker et al. 2008), the clinical signs of BRD remain consistent among the bacterial agents and can include appetite loss, fever, nasal discharge, depression, and respiratory distress (Griffin et al. 2010). Detection of BRD often relies heavily on the identification of these clinical signs by feedlot personnel, which has been reported to have poor sensitivity and specificity, potentially leading to misidentification of cattle that may require antimicrobial therapy (White and Renter 2009). Consequently, antimicrobial metaphylaxis, defined as the mass administration of antimicrobials to animals at high risk of BRD upon their arrival to a feedlot, is one of the most effective means of controlling BRD (Ives and Richeson 2015).

Because newly received feedlot cattle are at the highest risk of BRD (Griffin et al. 2010; Taylor et al. 2010), the bulk of the available literature characterizing the effectiveness of metaphylaxis follows animals through the first 40 to 120 days on feed (DOF), rather than the entire feeding period (Erickson et al. 2017; Credille et al. 2024; Johnson et al. 2024; Abi Younes et al. 2024a, 2024b). In this context, metaphylaxis has repeatedly been shown to decrease morbidity and mortality associated with BRD as well as improve growth performance indicators such as average daily gain (Erickson et al. 2017; Credille et al. 2024; Johnson et al. 2024; Abi Younes et al. 2024a, 2024b). Many of the studies that have followed calves through the end of the feeding period report similar decreases in morbidity and mortality, but do not report prevalence data of BRD associated pathogens (Tennant et al. 2014; Coppin et al. 2022; Horton et al. 2023).

In terms of the prevalence of the major bacterial pathogens, however, results are more heterogeneous. Credille et al. (2024) reported a decrease in the prevalence of *P. multocida* and no change in the prevalence of *M. haemolytica* after 14 DOF in stocker calves given tulathromycin. By contrast, Abi Younes et al. (2024b) reported a substantial decrease in the prevalence of *P. multocida* and a sustained increase in the prevalence of *M. haemolytica* from arrival to 13 DOF in cohorts given tulathromycin. Thus, questions remain about the efficacy of metaphylaxis, specifically in regard to decreases in bacterial pathogen prevalence, especially late in the feeding period. Therefore, the objective of this study was to quantify the effects of three different metaphylactic treatments on the nasal prevalence of *Pasteurellaceae* throughout the feeding period.

## Materials and methods

### Sample collection and handling

A total of 246 cattle at high risk of BRD were transported to the Texas Tech University Burnett Center near Idalou, TX as described previously by Coppin et al. (2022). Within 24 hours after arrival, steers were vaccinated against bovine rhinotracheitis virus, bovine viral diarrhea virus (types 1 and 2), bovine parainfluenza-3 virus, and bovine respiratory syncytial virus (Vista 5 SQ; Merck Animal Health, Kenilworth, NJ), clostridial pathogens (Vision 8 with Spur; Merck Animal Health), and *Mycoplasma bovis* (Myco-B ONE DOSE; American Animal Health, Inc., Grand Prairie, TX). Steers also received ivermectin (Vetrimec pour-on; Vet One; Boise, ID) for treatment of internal and external parasites. Treatments were applied the following day. Treatments consisted of the following: (1) negative control, 5 mL of sterile saline injected subcutaneously, (2) subcutaneous administration of florfenicol at 40 mg/kg of body weight; (3) subcutaneous administration of ceftiofur in the posterior aspect of the ear at 6.6 mg/kg of body weight; and (4) subcutaneous administration of tulathromycin at 2.5 mg/kg of body weight. Deep nasal swabs were collected from both nostrils using a 5-inch flocked swab in liquid Amies transport media. Samples were collected longitudinally at arrival, 30 DOF, and at the end of the feeding period (∼245 days on feed). Samples were stored at 4°C overnight prior to shipment to the University of Nebraska Veterinary Diagnostic Center in Lincoln, NE (UNLVDC).

Clinical health outcomes and therapeutic antimicrobial treatments for these steers have been reported previously by Coppin et al. (2022). Briefly, cattle were monitored daily for clinical signs of BRD throughout the feeding period and received therapeutic antimicrobial treatment when BRD case definitions were met. Metaphylaxis treatment affected BRD morbidity, with tulathromycin- and ceftiofur-treated cattle exhibiting less first-treatment BRD morbidity than control and florfenicol-treated cattle at arrival. In addition, the majority of BRD treatments occurred during the receiving period, with only 2 steers requiring BRD treatment during the finishing phase. Detailed morbidity, mortality, and treatment descriptions are reported elsewhere (Coppin et al. 2022).

### Bacteriological culture and MALDI-TOF MS

Each nasal swab was streaked onto tryptic soy agar with 5% sheep’s blood, chocolate agar, and MacConkey agar (Remel, Lenexa, KS). Plates were incubated for 18 to 24 h at 37°C and supplemented with 5% CO_2_ and examined at 24 and 48 h after incubation. Colonies with morphology consistent with *M. haemolytica, P. multocida*, and *H. somni* were selected for MALDI-TOF MS for definitive identification. For each isolate, a single colony was prepared in duplicate for direct smearing on a stainless-steel 96-well target plate according to the manufacturer’s instructions. The plate was then covered with α-cyano-4-hydroxycinnamic acid matrix (Bruker Daltonics, Billerica, MA) and set to dry. Spectra were obtained with a Microflex LT MALDI-TOF machine (Bruker Daltonics, Billerica, MA) set to automatic detection in positive linear mode with a range of 2 kDa to 20 kDa m/z and laser frequency of 60 Hz. The machine was calibrated for reference masses of 3637 to 16,952 Da using the manufacturer’s supplied bacterial test standard (Bruker Daltonics, Billerica, MA). The commercially available Biotyper software (Bruker Daltonics, Billerica, MA), used in conjunction with the manufacturer’s database (BDAL v.6 containing 6903 reference spectra) as well as a custom database from UNLVDC that contains additional profiles that were validated for inclusion, were used to identify bacterial isolates. Match scores were applied using manufacturer’s recommendations for genus and species level identifications.

### Data analysis

All analyses were performed in Stata 18.5 MP. Each sample was assigned the primary organism identification from MALDI-TOF MS. The identification was collapsed into the following mutually exclusive categories: *Pasteurellaceae, Moraxella*, others, and no isolation. Therefore, if *Moraxella* was identified in a sample as the primary organism by MALDI-TOF MS, then that sample was considered positive for *Moraxella*, and negative for the other three categories. We evaluated whether cattle positive to *Pasteurellaceae* at arrival were more likely to subsequently receive treatment for BRD by calculating the Odds Ratio (OR) and Fisher’s exact test. A multinomial logistic regression model was built to model the isolation frequency of bacteria (using the previously described collapsed categories) over sampling time and treatment. Interactions between metaphylaxis treatment, sampling event, and block were also assessed. Robust standard errors were used to adjust the variance for clustering at the animal level.

## Results

A total of 723 bacterial isolates were collected during this study. *Pasteurellaceae* were the most frequently isolated of the bacteria, representing 42.6% (*n* = 308) of the samples. *Moraxella* spp. were isolated in 24.48% (*n* = 177) of the samples, and bacteria classified as “others” were detected in 5.67% (*n* = 41) of samples. No potential bacterial pathogens (‘no isolation’) were found in 197 samples (27.25%).

There was no statistically significant association between arrival *Pasteurellaceae* isolation status and subsequent BRD treatment risk. The proportion of cattle treated for BRD did not differ between *Pasteurellaceae*-positive (36.5%) and *Pasteurellaceae*-negative (40.6%) animals (OR = 0.84 two-sided Fisher’s exact *P* = 0.59).

An overall metaphylaxis effect on bacterial isolation was observed (*P* < 0.01). Throughout the feeding period, no statistically significant difference between the experimental groups was observed for the nasal prevalence of *Moraxella* (*P* = 0.52). Upon arrival to the feedlot, steers assigned to the florfenicol group had the greatest nasal prevalence of *Pasteurellaceae* in comparison with the tulathromycin and control group (49% versus 31% and 32%, respectively; *P* < 0.05). After 30 DOF, no statistically significant differences between the control and experimental groups were observed in the nasal prevalence of *Pasteurellaceae*. Nonetheless, at one month on feed, florfenicol had a significantly greater percentage of ‘no isolation’ samples than ceftiofur (10% versus 2%; *P* < 0.05).

By the end of the feeding period, florfenicol and tulathromycin showed the lowest nasal prevalence of *Pasteurellaceae* (32% and 28%, respectively) and the difference between the two groups was not significant. Additionally, samples collected from steers receiving tulathromycin metaphylaxis had a significantly greater frequency of no bacterial isolation than the ceftiofur and control groups (59% versus 32% and 38%, respectively; *P* < 0.05). The florfenicol group also had a significantly greater frequency of no isolation than the ceftiofur group (53% versus 32%; *P* < 0.05). These trends are visualized in Fig. 1 and the fitted values for the multinomial logistic regression corresponding to *Pasteurellaceae* nasal prevalence (as percentages) are shown in Table 1.

**Figure 1:**
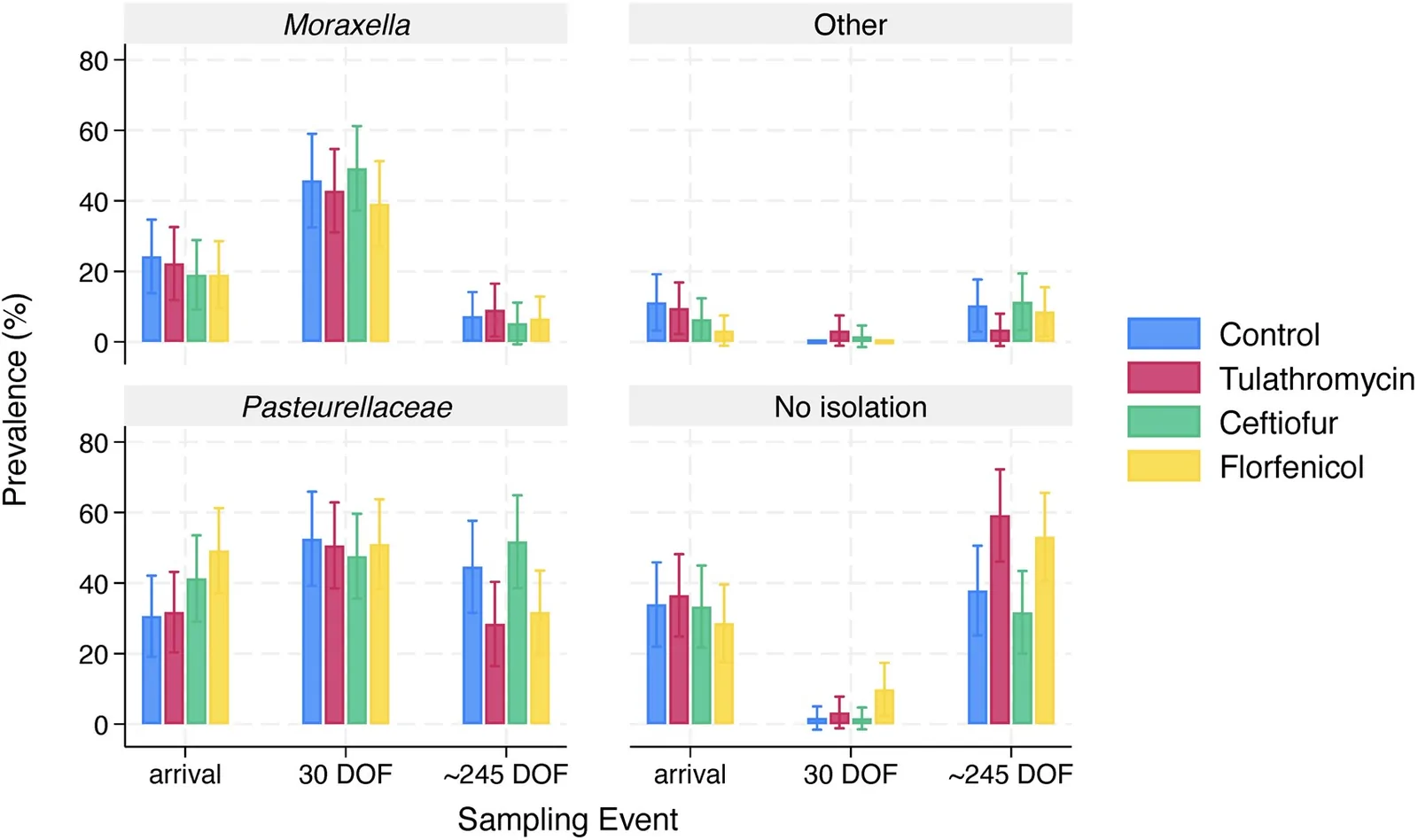
Nasal prevalence throughout the feeding period. Treatment groups are color coded. Subplots represent the nasal prevalence for *Pasteurellaceae*, *Moraxella*, other bacteria, and no bacterial isolation. Bars represent fitted values from a multinomial logistic regression (as percentages). Vertical lines represent 95% confidence intervals estimated using the delta method and therefore may extend beyond the natural range of prevalence (0% to 100%). DOF: days on feed.

**Table 1:** Nasal prevalence of *Pasteurellaceae* throughout the feeding period.

	Arrival	30 DOF	∼245 DOF
**Control**	31%^Aa^	53%^Ab^	45%^ABab^
**Tulathromycin**	32%^Aa^	51%^Ab^	28%^Aa^
**Ceftiofur**	41%^ABa^	48%^Aa^	52%^Ba^
**Florfenicol**	49%^Ba^	51%^Aa^	32%^Ab^

Values represent fitted values from a multinomial logistic regression model as percentages. Values in the same column with different uppercase superscripts differ statistically at a 0.05 significance level. Values in the same row with different lowercase superscripts differ statistically at a 0.05 significance level. DOF: days on feed.

## Discussion

The objective of the present study was to leverage the high diagnostic accuracy of MALDI-TOF MS to assess the effects of three different metaphylaxis treatment regimens on the nasal prevalence of *Pasteurellaceae*. The *Pasteurellaceae* family was chosen as a diagnostic target because three of the four bacterial pathogens associated with BRD (ie *Mannheimia haemolytica, Pasteurella multocida*, and *Histophilus somni*) are members of the *Pasteurellaceae* family. The *Moraxella* genu*s* was also included to assess the response of non-*Pasteurellaceae* organisms to metaphylactic treatment.

Antimicrobial metaphylaxis has long been recognized as one of the most effective methods of controlling BRD, particularly in high-risk cattle (Ives and Richeson 2015). Consistent with previous reports, we observed a metaphylaxis effect on the nasal prevalence of bacterial pathogens associated with BRD (Noyes et al. 2015; Credille et al. 2024). Our results also indicate that, despite the overall effectiveness of metaphylaxis, the proportion of samples positive for *Pasteurellaceae* increases between arrival and one month on feed in the tulathromycin group, similar to what was observed in the control group. Interestingly, an increase in the proportion of *Moraxella* isolations was also observed across all experimental groups between arrival and one month on feed. This is also consistent with previous studies, which report increased or similar rates of isolation for BRD pathogens after one month on feed for cattle receiving metaphylaxis (Noyes et al. 2015; Nobrega et al. 2021; Crosby et al. 2023; Abi Younes et al. 2024b). This would suggest that the primary driver of this trend is likely sampling time; specifically, the convergence of factors such as stress, commingling, and dietary changes that could promote bacterial colonization or invasion of the respiratory tract. Further research investigating respiratory tract colonization dynamics immediately following feedlot arrival is warranted.

Our results report that florfenicol and tulathromycin provided the best long-term decrease in the prevalence of *Pasteurellaceae*, achieving a 28% and 32% of positive samples, respectively. The ceftiofur group, by contrast, showed a sustained increase in the frequency of samples positive for *Pasteurellaceae*. This finding is very interesting, because studies comparing the efficacy of metaphylactic drugs using health outcomes and growth performance indicators (ie ADG, G: F) show little difference between ceftiofur and other drugs, such as tulathromycin (Word et al. 2020; Coppin et al. 2022; Pengpanun et al. 2026). Assessing the antimicrobial susceptibility of these isolates was beyond the scope of this study; however, previous work has shown widespread susceptibility of the major BRD pathogens to ceftiofur (Nobrega et al. 2021; Credille et al. 2024; Abi Younes et al. 2024a; Rattanapanadda et al. 2025), so ceftiofur resistance is an unlikely explanation for this trend. Microbiome shifts could have affected the *Pasteurellaceae* family in response to ceftiofur, but collecting such data was beyond the scope of this study. Furthermore, there is no available literature regarding the effects of ceftiofur metaphylaxis on the nasopharyngeal microbiome of cattle. Our data indicate that the effects of ceftiofur may have a shorter duration of effectiveness, but further research is required to disentangle these findings.

While this study significantly advanced our understanding of the long-term effects of metaphylactic treatments in decreasing the nasal prevalence of *Pasteurellaceae*, there were some limitations. While the diagnostic accuracy of MALDI-TOF has been well characterized (Puchalski et al. 2016; Van Driessche et al. 2019), we chose to collapse bacterial identifications of the isolates to improve model convergence, thereby losing the ability to evaluate the effect of the metaphylactic drugs at the species level. Selecting one isolate per sample, while common in diagnostic workflows, introduces the possibility of culture bias. Additionally, the cattle in this study were not exposed to the environmental stressors typically present in large commercial feedlots. Another important consideration when interpreting these findings is that clinical morbidity and subsequent therapeutic antimicrobial use could also influence nasopharyngeal bacterial ecology. In the companion study using the same steers, BRD morbidity differed among metaphylaxis groups and steers that developed clinical disease received additional antimicrobial therapy (Coppin et al. 2022). Consequently, changes in bacterial isolation frequency observed later in the feeding period reflect the combined effects of arrival metaphylaxis and subsequent therapeutic interventions (the total effect). Because the present study was not designed to evaluate the direct effects of individual therapeutic treatments on bacterial shedding, these factors could not be fully separated. Future studies should evaluate longitudinal relationships among BRD morbidity, therapeutic antimicrobial administration, and respiratory pathogen carriage.

## Conclusion

In conclusion, this study provides important insights into the effects of the metaphylactic use of florfenicol, tulathromycin, and ceftiofur on BRD pathogen nasal prevalence throughout the feeding period, either directly or by influencing subsequent antimicrobial treatments. Our findings indicate that tulathromycin and florfenicol metaphylactic use produces sustained decreases in *Pasteurellaceae* through the end of the feeding period. Future research should incorporate pathogen quantification and microbiome analyses to more comprehensively characterize the ecological and population-level effects of antimicrobial metaphylaxis, thereby improving our understanding of its impact on animal health and antimicrobial stewardship.
